# Simple Whole-Mount Staining Protocol of F-Actin for Studies of the Female Gametophyte in Agavoideae and Other Crassinucellate Ovules

**DOI:** 10.3389/fpls.2020.00384

**Published:** 2020-04-09

**Authors:** Alejandra G. González-Gutiérrez, Jorge Verdín, Benjamín Rodríguez-Garay

**Affiliations:** ^1^Unidad de Biotecnología Vegetal, CIATEJ, Centro de Investigación y Asistencia en Tecnología y Diseño del Estado de Jalisco, A.C., Zapopan, Mexico; ^2^Unidad de Biotecnología Industrial, CIATEJ, Centro de Investigación y Asistencia en Tecnología y Diseño del Estado de Jalisco, A.C., Zapopan, Mexico

**Keywords:** double fertilization, ovular apparatus, central cell nucleus, cytoskeleton, F-actin staining, fixed-tissue staining, confocal microscopy

## Abstract

During plant sexual reproduction, F-actin takes part in the elongation of the pollen tube and the movement of sperm cells along with it. Moreover, F-actin is involved in the transport of sperm cells throughout the embryo sac when double fertilization occurs. Different techniques for analysis of F-actin in plant cells have been developed: from classical actin-immunolocalization in fixed tissues to genetically tagged actin with fluorescent proteins for live imaging of cells. Despite the implementation of live cell imaging tools, fixed plant tissue methods for cytoskeletal studies remain an essential tool for genetically intractable systems. Also, most of the work on live imaging of the cytoskeleton has been conducted on cells located on the plant’s surface, such as epidermal cells, trichomes, and root hairs. In cells situated in the plant’s interior, especially those from plant species with thicker organ systems, it is necessary to utilize conventional sectioning and permeabilization methods to allow the label access to the cytoskeleton. Studies about the role of F-actin cytoskeleton during double fertilization in plants with crassinucellate ovules (e.g., *Agave, Yucca, Polianthes, Prochnyantes*, and *Manfreda*) remain scarce due to the difficulties to access the female gametophyte. Here, we have developed a straightforward method for analysis of F-actin in the female gametophyte of different Agavoideae sub-family species. The procedure includes the fixation of whole ovules with formaldehyde, followed by membrane permeabilization with cold acetone, a prolonged staining step with rhodamine-phalloidin, and Hoechst 33342 as a counterstain and two final steps of dehydration of samples in increasing-concentration series of cold isopropanol and clarification of tissues with methyl salicylate. This technique allows the analysis of a large number of samples in a short period, cell positioning relative to neighbor cells is maintained, and, with the help of a confocal microscope, reconstruction of a single 3D image of F-actin structures into the embryo sac can be obtained.

## Introduction

The actin cytoskeleton is a complex structure present in all eukaryotic cells (Povarova et al., 2012). In plants, actin is an important research target since it is involved in key cellular processes such as cell polarity, division plane determination, organogenesis, and intracellular signaling (Higaki et al., 2007). During reproduction of higher plants, actin filaments also play an important role; they are involved in pollen tube elongation (Vidali et al., 2001), vesicle and organelle transport (Drøbak et al., 2004; Cai and Cresti, 2009) and self-incompatibility responses (Roldán et al., 2012). Moreover, actin is involved in the female gametophyte development (Huang et al., 1999; Kawashima and Berger, 2015), in double fertilization (Huang and Sheridan, 1998; Kawashima et al., 2014) and the subsequent processes of endosperm (Świerczyńska and Bohdanowicz, 2003; Barranco-Guzmán et al., 2019) and embryo development in the seed (Kimata et al., 2016).

Due to its relevance, different techniques for visualization and analysis of F-actin have been developed: from classical actin-immunolocalization (Lazarides and Weber, 1974; Andersland et al., 1994) and phalloidin-based labeling (Wulf et al., 1979; Vandekerckhove et al., 1985) in fixed tissues, to genetically tagged actin with fluorescent proteins for live imaging (Kost et al., 1998). Among the latter, Lifeact, a short peptide consisting of the first 17 amino acids of *Saccharomyces cerevisiae* Abp14p, has revolutionized the study of F-actin physiology in eukaryotic cells (Sheahan et al., 2004; Era et al., 2009). Despite such progress, live-cell imaging is limited to genetically tractable systems. Also, most cytoskeleton’s live imaging in plants has been conducted on surface cells (Blancaflor and Hasenstein, 2000) such as pollen tubes (Cheung et al., 2008), trichomes (Chang et al., 2019), and root hairs (McCurdy and Gunning, 1990; Colling and Wasteneys, 2005). However, the study of some biological processes, such as female gametophyte development and fertilization, requires the observation of the interior of the plant, which has specific technical challenges (Blancaflor and Hasenstein, 2000; Cheng, 2006).

The major technical challenge for female gametophyte imaging studies is the thickness of the sporogenous layers that cover it (Schneitz et al., 1995). These layers of nucellar tissue lead to poor quality observations or even access prevention of chemical and immunological dyes to their targets. The latter is particularly true for crassinucellate ovules (e.g., *Agave, Yucca, Polianthes, Prochnyantes*, and *Manfreda*) (Rudall, 1997), where one or more layers of hypodermic tissues are found between the meiocyte and the apex of the nuclei (Reddy, 2007; Endress, 2011). A first choice to solve this problem is two-photon confocal microscopy (Diaspro and Robello, 2000; Feijó and Moreno, 2004; Kimata et al., 2016) or, a cheaper alternative, microtome sectioning (Stelly et al., 1984). However, in microtomy techniques, the positioning of cells concerning neighbor cells are often lost, and the resulting sectioned planes are difficult to reconstruct in a single three-dimensional (3D) image (Haseloff, 2003; Barrell and Grossniklaus, 2005). Tissue permeabilization and clearing is an option to overcome those obstacles (Cheng, 2006). Under this strategy, thick tissue masses are made translucent through chemical treatments with substances with a high refractive index such as xylene, chloral hydrate, and methyl salicylate (Herr, 1993), reducing the problems of light scattering and spherical aberration, allowing high image resolution (Haseloff, 2003).

Here, we report an improved whole-mount technique to label F-actin in the female gametophyte of thick crassinucellate ovules of some genera of the Agavoideae sub-family and *Petunia hybrida* (Rezanejad, 2008) as an example of a different plant family. This technique combines classical tissue fixation, chemical staining, and a tissue clarification step that significantly improves image quality. This protocol allows the analysis of a large number of samples in a short period, cell positioning relative to neighboring cells is maintained, and 3D images of the cytoskeleton in deep tissues can be obtained.

## Materials and Equipment

### Reagents

PIPES, 1,4-piperazinediethanesulfonic acid (Sigma, Cat. No. P1851)

EGTA, Ethylene glycol-bis(2-aminoethylether)-N,N,N′,N′-tetraacetic acid (Sigma, Cat. No. E3889)

Magnesium chloride hexahydrate (Sigma, Cat. No. M2670)

Potassium hydroxide (Sigma, Cat. No. 221473)

37% Formaldehyde solution (Sigma, Cat. No. 252549)

Acetone (Sigma, Cat. No. 270725)

BSA, bovine serum albumin fraction V (Sigma, Cat. No. 10735078001)

Rhodamine-phalloidin (Molecular Probes, Cat. No. R415)

Hoechst 33258 pentahydrate (Molecular Probes, Cat. No. H21491)

2-Propanol (Sigma, Cat. No. 190764)

Methyl salicylate (Sigma, Cat. No. M6752)

Leica immersion oil type F (Leica, Cat. No. 11513859)

Latrunculin B from *Latruncula magnifica* (Sigma, Cat. No. L5288).

### Materials

0.2–0.6 ml microcentrifuge tubes

Glass Pasteur pipettes and bulbs

Insulin needles and syringes

Glass slides, 75 mm × 25 mm (Corning, Cat. No. 2947)

Glass coverslips, 24 mm × 40 mm (Thermo Fisher Scientific, Cat. No. C7931)

Straight fine point tweezers.

### Equipment

TCS SPE Confocal microscope (Leica Microsystems)

EZ4 HD Dissecting stereomicroscope (Leica Microsystems)

LAS X software^®^ (Leica Microsystems).

## Solutions Recipes

*ASB (Actin-stabilizing buffer)* (Płachno and Świa̧tek, 2012)

50 mM PIPES, 10 mM EGTA, and 1 mM MgCl_2_, pH 6.8 adjusted with 10M KOH. It is important to previously dissolve EGTA and PIPES in a few drops of 10M KOH.

Fixative solution

3.7% formaldehyde in ASB. It is preferable to use the fixative solution just after preparation; however, it can be stored at 4°C for up to 5 days.

Blocking solution

1% BSA in ASB. BSA solution can be stored at 4°C.

Rhodamine-phalloidin stock solution

6.6 μM rhodamine-phalloidin in methanol. Store the solution at −20°C in darkness.

Hoechst 33258 pentahydrate stock solution

10 mg/ml Hoechst 33258 pentahydrate in distilled water. Prepare 2 ml aliquots, store them protected from light at −20°C.

Latrunculin B stock solution

20 μM latrunculin B in ethanol. Store at −20°C in darkness.

## Methods

### Sample Collection

**FIGURE 1:**
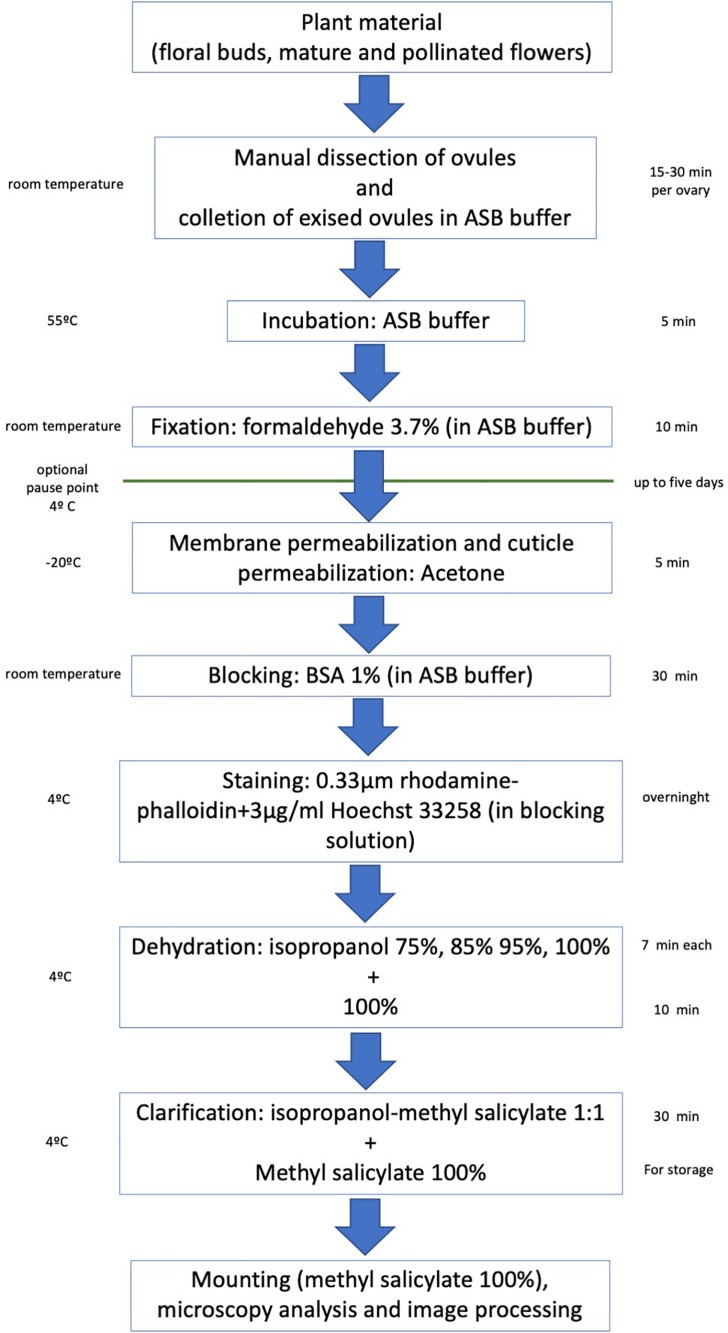
Overview of the main steps of the rhodamine-phalloidin staining and methyl salicylate clarification of crassinucellate ovules.

Flower buds of different sizes, mature flowers and immature fruits (collected at a distinct time after pollination) are collected and processed as follows to visualize the F-actin cytoskeleton at different stages of the female gametophyte development and early embryogenesis (an overview of the protocol described below is shown in Figure 1).

### Dissection of Ovules

The dissection of ovules and immature seeds is performed with the help of straight fine-point tweezers and an insulin needle under the stereoscope.

### Ovules Collection and Fixation

Ovules of the same ovary are collected in a 0.2–0.6 ml microtube containing ASB (N.B.1) at 25°C (room temperature). Once enough ovules have been collected (keep in mind that a fraction of ovules is lost during the staining process), they are incubated in ASB at 55°C for 5 min (N.B.2). Afterward, ovules are fixed with a fixative solution for 7–10 min at 25°C. Small-sized ovules require less fixation time than larger ones (e.g., *Agave* ovules are fixed for 10 min, while *Petunia* ovules are fixed for 7 min). After fixation, rinse ovules twice with ASB. If needed, previously fixed and washed ovules can be stored up to five days at 4°C protected from light. Afterward, continue the technique in section “Cuticle Solubilization and Membrane Permeabilization.”

N.B.1 unlike similar protocols, we have used ASB instead of MBS. EGTA contained in ASB binds Ca^2+^ ions, which prevents actin filaments severing (Yin et al., 1981; Hepler, 2016).

N.B.2 pretreatment at 55°C allows more efficient fixative penetration. For Asparagales species, warm buffer incubation does not affect the structure of neither the ovule nor actin filaments.

### Cuticle Solubilization and Membrane Permeabilization

After completing the fixation step, quickly rinse twice ovules with acetone at −20°C, and afterward keep them in fresh cold acetone for 5 min. Finally, wash ovules three times with ASB or until it remains crystalline.

### Blocking and Staining

Pre-incubate ovules in blocking solution (1% BSA in ASB) for 20 min at room temperature. Then, stain ovules overnight at 4°C with 0.33 μM rhodamine-phalloidin for labeling F-actin and 3 μg/ml Hoechst 33258 to counterstain cell nuclei (diluted in blocking solution).

### Dehydration

After the staining period (N.B.3), dehydrate ovules in isopropanol (N.B.4) increasing-concentration solutions (75, 85, 95, and 100%) for 7 min each at 4°C, and finally, in 100% isopropanol for 10–12 min also at 4°C. All isopropanol solutions must be continuously renewed, and samples should be gently shaken to homogenize the exposure of tissues to isopropanol.

N.B.3 it is not necessary to wash the stain with a buffer since the next dehydration series work also as a washing step.

N.B.4 in this protocol we use isopropanol instead of ethanol or methanol to dehydrate samples since dehydration with isopropanol is faster and produces better quality images.

### Clarification

For tissue clarification, remove isopropanol and add a 1:1 methyl salicylate-isopropanol solution for 30–60 min. In the beginning, ovules will remain on top of the solution, but eventually, they will sink to the bottom of the microcentrifuge tube. Incubation time in methyl salicylate-isopropanol concludes when all ovules precipitate. Before observation, ovules are incubated in 100% methyl salicylate for at least 30 min. During this time, ovules get completely clear. Ovules can be kept in this solution in darkness at 4°C for about a week.

### Mounting and Microscopy

Mount treated ovules directly on glass slides with 100% methyl salicylate. Observe the samples under the confocal microscope using a 532 nm laser for rhodamine-phalloidin (ex/em = 540/556 nm) and a 405 nm laser for Hoechst 33258 observation (ex/em = 352/461 nm). Analyze images with LAS X^®^ software or any other appropriate software.

### Control of the Specificity of Rhodamine-Phalloidin F-Actin Staining

To confirm the specificity of rhodamine-phalloidin F-actin staining, ovules of *Agave* sp. were treated with latrunculin-B, which prevents G-actin polymerization (Spector et al., 1983). Inhibition assays were conducted following the protocol of Yuan et al. (2002) with some modifications; in short, dissected ovules of *Agave* sp. were collected in microcentrifuge tubes containing culture medium (5 mM HEPES, 1 mM KCl, 1 mM MgCl_2_, 0.1 mM CaCl_2_, 3% w/v sucrose). Once enough ovules were collected, they were incubated in culture medium with (20 nM, final concentration) or without (control) latrunculin B for 4 h, at 22°C. After completing this incubation, ovules were quickly washed three times with culture medium and, finally, fixed and stain-cleared as described above.

## Results

**FIGURE 2:**
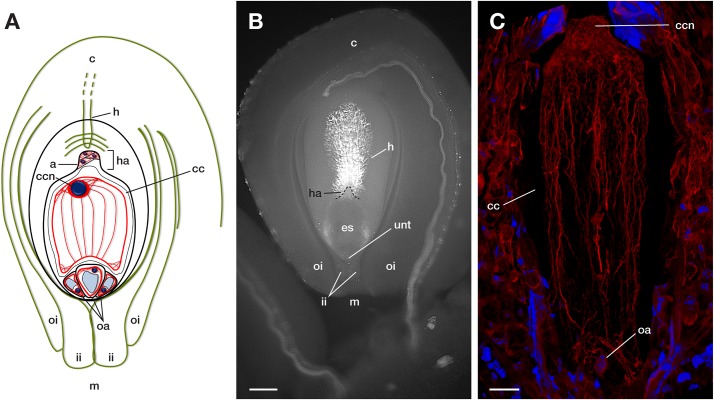
Main components of a mature crassinucellate ovule of the Agavoideae sub-family. **(A)** Schematic representation of an anatropous, bitegmic, crassinucellate ovule of *Agave*. **(B)** Representative microphotography and **(C)** F-actin cytoskeleton of a mature embryo sac of *Agave.* c, chalaza; m, micropyle; oi, outer integument; ii, inner integument; unt, uniseriate nucellar tissue; es, embryo sac; h, hypostase; ha, haustorium; oa, ovular apparatus; a, antipodal cells; cc, central cell; ccn, central cell nucleus. The blue color in A and C indicate nuclei of cells (Hoechst 33258) and red color (phalloidin) represents F-actin filaments. Bar in **(B)**, 90 μm, in **(C)**, 40 μm.

**FIGURE 3:**
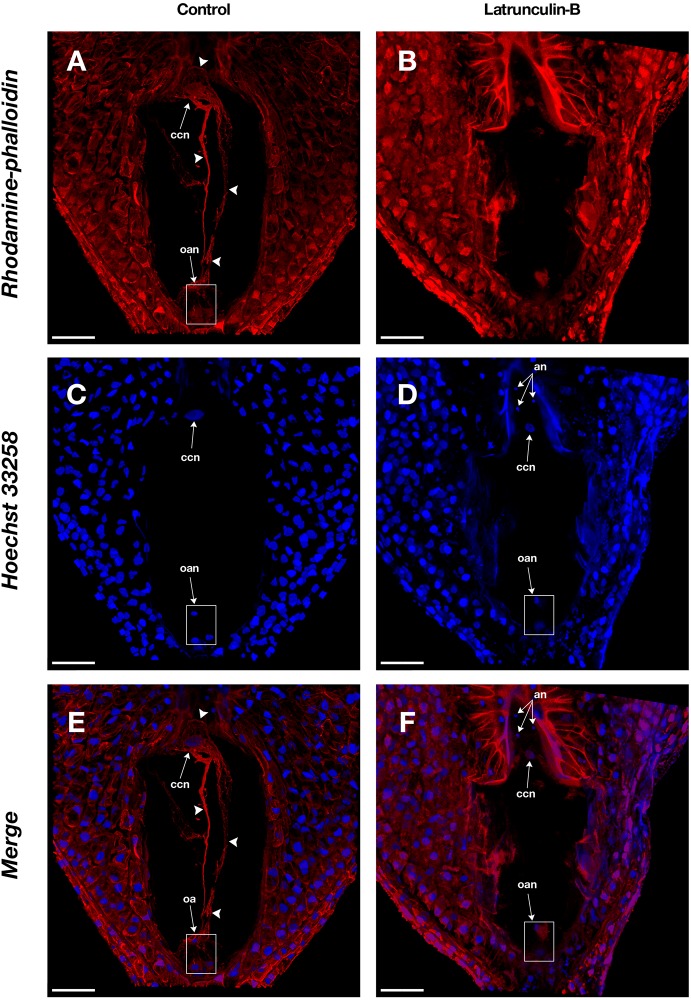
Actin filaments in the embryo sac of *Agave* sp. **(A,C,E)** and the effect of latrunculin B **(B,D,F)**. **(A,B)** show rhodamine-phalloidin staining of the control and latrunculin B treated cells, respectively. **(C,D)** show Hoechst 33258 counterstaining. **(E,F)** are the merge of rhodamine-phalloidin and Hoechst 33258 channels. Arrowheads indicate F-actin filaments and cables that form part of the cytoskeleton of each cell type in the female gametophyte. an, antipodal nuclei; oan, ovular apparatus nuclei; oa, ovular apparatus; ccn, central cell nucleus. Bars, 40 μm.

**FIGURE 4:**
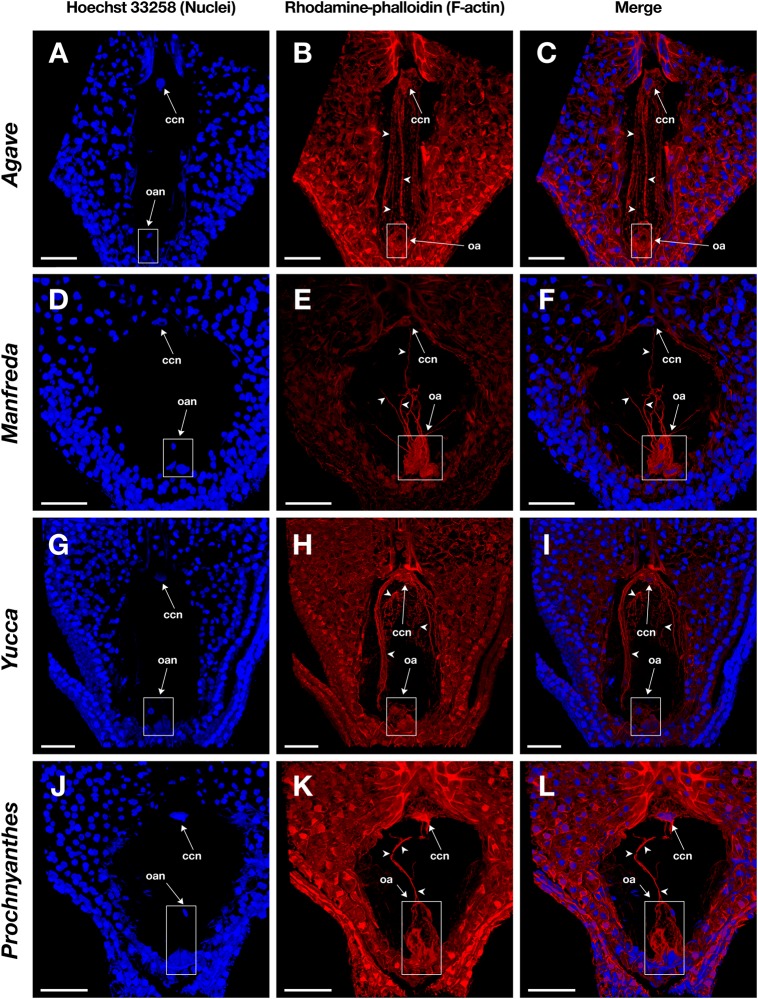
Mature embryo sacs of four different species belonging to the Agavoideae sub-family stained with Hoechst 33258 (left column) and rhodamine-phalloidin (central column), and clarified with methyl salicylate. **(A–C)** *Agave tequilana*; **(D–F)** *Manfreda elongata*; **(G–I)** *Yucca* sp.; and, **(J–L)** *Prochnyanthes* sp. Arrowheads indicate F-actin filaments and cables that form part of the cytoskeleton of each cell type in the female gametophyte. ccn, central cell nucleus; oan, ovular apparatus nuclei; oa, ovular apparatus. Bars, 40 μm.

**FIGURE 5:**
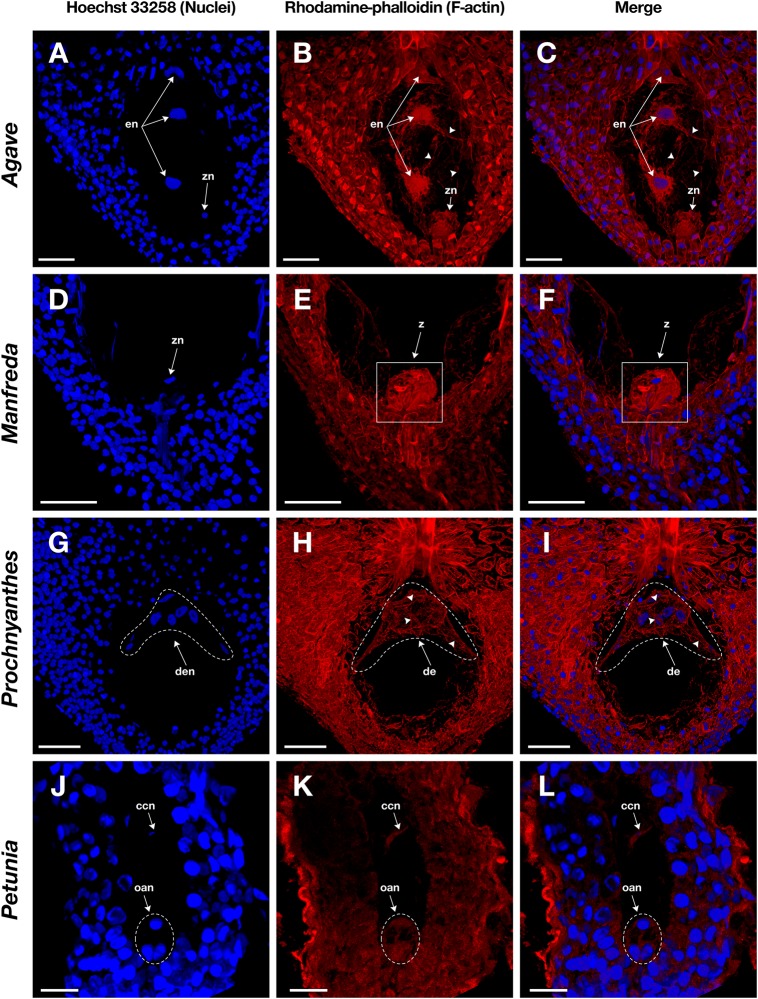
Female gametophyte of four different species with crassinucellate ovules stained with Hoechst 33258 (left column) and rhodamine-phalloidin (central column), and clarified with methyl salicylate. **(A–C)** Endosperm nuclei of an immature seed of *Agave tequilana*; **(D–F)** zygote of *Manfreda elongata*; **(G–I)** chalazal endosperm chamber of *Prochnyanthes* sp.; and **(J–L)** mature embryo sac of *Petunia hybrida*. Arrowheads indicate F-actin filaments and cables that form part of the cytoskeleton inside the embryo sac. en, endosperm nuclei; z, zygote; zn, zygote nucleus; de, developing endosperm; den, developing endosperm nuclei; ccn, central cell nucleus; oan, ovular apparatus nuclei; oa, ovular apparatus. Bars, 40 μm.

The protocol described here can be performed in 48 h, which includes an overnight staining incubation (Figure 1). It enables us to perform microscopy observations of whole embryo sacs and determine the 3D allocation of the F-actin cytoskeleton inside them (Figures 2, 3A,C,E, 4, 5). Up to 25–30 μm thick ovules could be observed without microtome sectioning (Supplementary Movie 1).

Fixation time should be optimized for each plant species and the sample developmental stage. In general, smaller ovules and ovules in early development stages need shorter fixation times. Cuticle solubilization and dehydration steps are also critical; they require constant solutions renewal and gentle hand-shaking to homogenize components. Rhodamine-phalloidin and Hoechst 33258 fluorophores maintain their fluorescence stable up to 10 days on samples treated with this stain-clearing technique when they are stored at 4°C. Moreover, co-staining with Hoechst 33258 provides information on the spatial position of nuclei within the cell and its relationship with actin filaments (Figures 4C,F,I,L, 5C,F,I,L).

Latrunculin B inhibition assays were performed on *Agave* embryo sacs (Figure 3) to confirm the specificity of rhodamine-phalloidin F-actin staining. In the presence of latrunculin B, actin filaments appeared fragmented or completely disappeared (Figures 3B,D,F) while in the control treatment, intact actin filaments were observed (Figures 3A,C,E).

The methyl salicylate clearing step is critical for the protocol’s success since it allows us to get over the physical barriers that usually impede imaging of the whole embryo sac. Sample observation needs a minimum of 30 min incubation in methyl salicylate after mounting; longer incubation times usually improve image quality.

Following this protocol, we managed to visualize the F-actin cytoskeleton in the female gametophyte of different genera of the Agavoideae sub-family (*Agave, Manfreda, Yucca*, and *Prochnyanthes*) (Figures 4, 5A–I) and other non-related species with crassinucellate ovules such as *P. hybrida* (Figures 5J–L).

This protocol is useful for the analysis of different female developmental stages of crassinucellate ovules, from the differentiation of the megaspore mother cell, the megasporogenesis, megagametogenesis, and the double fertilization, to early stages of embryo and endosperm development (Figures 4, 5). The rhodamine-phalloidin staining followed by methyl salicylate clarification allows identifying dense actin cables as well as thin actin filaments (Figures 4B,E,H,K, 5B,E,H,K).

In the mature embryo sac, the cytoskeleton of each cell type (central cell, synergid, antipodal cells, and egg cell) located beneath the membrane could be observed; similarly, the F-actin coat around the nuclei of the cells could be appreciated with great detail (Figures 4, 5). F-actin strands that run parallel along the chalazal-micropylar axis of the large central cell vacuole were detected without spherical aberration. This technique allowed the transmission of the microscope laser through the thicker tissues that are found in the immature seeds; thus, actin cables that connect free nuclei of the endosperm in the embryo sac could be registered (Figures 5A–C, G–I).

## Discussion

Despite the great progress of fluorescent protein-tagging of cellular targets for live-cell imaging, phalloidin conjugated with any fluorochrome remains the gold standard for actin filament visualization (Melak et al., 2017). Immunofluorescence- and phalloidin-based techniques are useful for the structural analysis of cytoskeleton, especially in fixed cells, and, even when they share some critical steps like fixation and permeabilization (Blancaflor and Hasenstein, 2000), each one presents its advantages and drawbacks.

Some researchers have shown that phalloidin, fluorescent proteins and antibodies give different imaging results (Tang et al., 1989; Le et al., 2003; Thomas et al., 2009; Zhang et al., 2018; Flores et al., 2019). They claim that phalloidin and fluorescent proteins may induce actin bundles artifacts (Le et al., 2003); nevertheless, others suggest those actin forms are biologically active and produced by specific actin associated proteins (Bartles, 2000; Thomas et al., 2009; Zhang et al., 2018). In this work, we did not observe actin bundles in surface cells stained with phalloidin (data not shown), which were subjected to exactly the same staining conditions as embryo sacs, where actin bundles are abundant (Figures 3A, 4B,E,H). The latter suggests that bundles are produced in specific cellular contexts and they are not phalloidin induced artifacts.

Perhaps one of the main perks of immunolabeling is the possibility of applying two or more antibodies on the same sample to co-label several proteins (Shimamura, 2015) (e.g., actin and microtubules). Despite the later, antibodies are generally large; therefore, a proper fixation, membrane permeabilization, and cell wall digestion result critical for the successful diffusion of antibodies, especially into deeper cell layers (Pasternak et al., 2015), as is the case with the female gametophyte. On the other hand, the relatively small size of phalloidin derivatives might be helpful in its permeation through the cell wall and membrane of plant cells. Thus, in the present protocol, permeabilization with detergents like DMSO or Triton X100, and degradation of the cell wall with enzymes were not necessary, which contribute to shorten the duration of the technique.

In this improved method, incubation, fixation, permeabilization, and clarification were performed in ASB to stabilize F-actin, after the successful experience of Płachno and Świa̧tek (2012). They managed to stain the actin filaments in extra-ovular embryo sacs of *Utricularia nelumbifolia* (Płachno and Świa̧tek, 2012). ASB contains EGTA, which binds Ca^2+^ ions that prevents actin filaments severing (Yin et al., 1981; Hepler, 2016).

Due to the intrinsic features of plant cells and tissues -like cell walls, vacuoles, and cuticle layers- most of the imaging work has been conducted on plant’s surface cells. If inside cells need to be observed, microtome sectioning used to be the approach. This technique is time-consuming and provides images in only two dimensions (Haseloff, 2003). Here, by using methyl salicylate to clarify tissues, we accomplished the imaging of the complete F-actin cytoskeleton within the embryo sac (Figures 3–5), a highly vacuolated structure that is located inside the ovule and surrounded by one or more layers of nucellar tissue (Figure 2).

Methyl salicylate has been successfully used in the structural analysis of the female gametophyte development of *Solanum* (Stelly et al., 1984) and *Polianthes* (González-Gutiérrez and Rodríguez-Garay, 2016). Nevertheless, according to Richardson and Lichtman (2015), organic solvent-based clearing methods, which remove water from the cell, affect the capacity of fluorophores to maintain its emission. Despite those remarks, in our studies, the employment of methyl salicylate to clarify tissues did not interfere in the detection and quality of the fluorescent label.

Overall, this improved rhodamine-phalloidin staining followed by methyl salicylate clearing of whole ovules represents an option for the study of F-actin cytoskeleton in plant species where tagging with fluorescent proteins is not feasible. This approach is especially useful for imaging thick crassinucellate ovules, which till this report has not been successfully labeled and imaged (Escobar-Guzmán et al., 2015). Moreover, this technique could be useful as a first, easy, and rapid approach to visualize the actin cytoskeleton of the female gametophyte of different plant species.

## Data Availability Statement

The datasets generated for this study are available on request to the corresponding author.

## Author Contributions

AG-G carried out the microscope analyses, the acquisition, and interpretation of images and drafted the manuscript. JV helped with interpretation of data and drafted the manuscript. BR-G conceived and coordinated the study, carried out the analysis and interpretation of the data, and drafted the manuscript. All authors read and approved the final manuscript.

## Conflict of Interest

The authors declare that the research was conducted in the absence of any commercial or financial relationships that could be construed as a potential conflict of interest.

## Supplementary Material

MOVIE S1 Animation of a 3D-projection of the micropylar region of an embryo sac of Agave spp stained with rhodamine-phalloidin. Note that F-actin bundles are conspicuosly stained.
